# Discovery and implementation of a novel pathway for *n*-butanol production via 2-oxoglutarate

**DOI:** 10.1186/s13068-019-1565-x

**Published:** 2019-09-30

**Authors:** Sofia Ferreira, Rui Pereira, Filipe Liu, Paulo Vilaça, Isabel Rocha

**Affiliations:** 1 CEB-Centre of Biological Engineering, University of Minho, Campus de Gualtar, 4710-057 Braga, Portugal; 2 SilicoLife Lda, Rua do Canastreiro 15, 4715-387 Braga, Portugal; 3 Present Address: Department of Biology and Biological Engineering, Chalmers University of Technology, 412 96 Gothenburg, Sweden; 4 Present Address: Mathematics and Computer Science Division, Argonne National Laboratory, Argonne, IL USA; 5 Instituto de Tecnologia Química e Biológica António Xavier, Universidade Nova de Lisboa (ITQB-NOVA), Oeiras, Portugal

**Keywords:** *n*-Butanol, *E. coli*, Metabolic engineering, Enumeration algorithms, 2-Oxoglutarate

## Abstract

**Background:**

One of the European Union directives indicates that 10% of all fuels must be bio-synthesized by 2020. In this regard, biobutanol—natively produced by clostridial strains—poses as a promising alternative biofuel. One possible approach to overcome the difficulties of the industrial exploration of the native producers is the expression of more suitable pathways in robust microorganisms such as *Escherichia coli*. The enumeration of novel pathways is a powerful tool, allowing to identify non-obvious combinations of enzymes to produce a target compound.

**Results:**

This work describes the in silico driven design of *E. coli* strains able to produce butanol via 2-oxoglutarate by a novel pathway. This butanol pathway was generated by a hypergraph algorithm and selected from an initial set of 105,954 different routes by successively applying different filters, such as stoichiometric feasibility, size and novelty. The implementation of this pathway involved seven catalytic steps and required the insertion of nine heterologous genes from various sources in *E. coli* distributed in three plasmids. Expressing butanol genes in *E. coli* K12 and cultivation in High-Density Medium formulation seem to favor butanol accumulation via the 2-oxoglutarate pathway. The maximum butanol titer obtained was 85 ± 1 mg L^−1^ by cultivating the cells in bioreactors.

**Conclusions:**

In this work, we were able to successfully translate the computational analysis into in vivo applications, designing novel strains of *E. coli* able to produce *n*-butanol via an innovative pathway. Our results demonstrate that enumeration algorithms can broad the spectrum of butanol producing pathways. This validation encourages further research to other target compounds.

## Background

Butanol poses as a promising alternative to ethanol as a fuel due to its superior properties, such as higher energy content, less corrosiveness and higher blending capacity with gasoline [1]. *N*-Butanol is natively produced together with ethanol and acetone through acetone–butanol–ethanol (ABE) fermentation by *Clostridium* species [2, 3]. Engineering its native host to improve butanol production is often complicated by, among other factors, the difficulty in performing genetic manipulations in *Clostridium* spp. and the formation of spores during butanol production in the solventogenic phase.

The expression of *Clostridia* butanol pathway in a more robust microbial host, such as *Escherichia coli* was firstly implemented by the Atsumi et al. in 2008 [4]. Nevertheless, the low butanol titers obtained with *E. coli* show that many challenges remain in turning this organism into a viable butanol production host. For example, the first reaction of the clostridial pathway—the condensation of two molecules of acetyl-CoA into one molecule of acetoacetyl-CoA—is thermodynamically unfavorable [5]. Also, other enzymes of this pathway appear to be poorly expressed in *E. coli*, such as butyryl-CoA dehydrogenase, the enzyme that catalyzes the reduction of butanoyl-CoA into crotonoyl-CoA [6]. Finally, the high requirement of reducing power and the consequent competition with native fermentation pathways have also to be considered when optimizing butanol production in heterologous hosts [7]. Several rational strategies were tested in *E. coli* to increase butanol productivity, especially metabolic engineering approaches such as manipulation of central carbon metabolism, elimination of competing pathways, gene overexpression, cofactor balancing, expression of novel enzymes and consumption of different substrates [8].

A strategy to tackle some of the issues arisen from expressing clostridial enzymes is to identify alternative butanol biosynthetic pathways more suitable for expression in standard microbial production hosts. Until now, only three alternative pathways for producing butanol have been tested in *E. coli,* besides the clostridial one. One explores the keto-acids metabolism [9] and the other two explore an engineered version of the β-oxidation pathway present in *E. coli* [10, 11]. Regarding other microorganisms, a pathway converting malonyl-CoA into butanol in cyanobacteria was also reported [12].

Shen and Liao took advantage of the native amino acid pathway overcoming the need to involve CoA-dependent intermediates and producing simultaneously butanol and propanol [9]. In their work, *E. coli* was engineered to increase the pool of 2-ketobutyrate, a common keto-acid intermediate in isoleucine biosynthesis, later converted into butanol through the norvaline biosynthetic pathway.

Dellomonaco and coworkers engineered *E. coli* to activate the reverse β-oxidation cycle in the absence of the inducing substrate (fatty acids) [10]. The constitutive expression of this pathway was achieved by introducing mutations in the corresponding transcriptional regulators (*fad*, *ato* and *crp*) and knocking out *arcA*. In a similar approach, Debabov et al. also explored the reversed β-oxidation pathway, but only expressing enzymes from this pathway and an aerotolerant mutant *adhE* to convert butyryl-CoA into butanol [11].

In this work, we used a previously optimized hypergraph algorithm [13] to enumerate all possible pathways leading from *E. coli*’s metabolism to butanol. The pathways were analyzed to infer their feasibility to be applied in vivo. First, the solutions were evaluated according to diverse criteria (butanol yield, number of reactions and novelty) to seek the most promising solutions. For each of the steps composing the novel pathway, enzymes were selected considering curated gene sequences and the experimental data available in the literature. Here, novel strains of *E. coli* producing butanol through a novel pathway were developed. The resulting set of genes catalyzing the successive reactions from 2-oxoglutarate to butanol originates from diverse microorganisms.

## Results

### Computational analysis and manual curation of heterologous pathways

The set of heterologous pathways generated using our methodology described in [13] included 105,954 alternatives. After discarding all the pathways that could not carry flux into the target product (butanol), only 40,608 different pathways were left. Since the clostridial butanol pathway possesses 6 heterologous reactions, a competing alternative should not be much larger. Therefore, the dataset was filtered to include only pathways with a maximum of 7 heterologous reactions. The application of this filter resulted in 316 pathways, which, in their turn, were ranked by size: 4 pathways with size equal to three reactions; 4 pathways with size equal to four reactions; 120 pathways with size equal to six reactions and 188 pathways with size equal to seven reactions. Since a higher number of catalytic steps do not always translate into a higher number of expressed proteins, we have also checked pathways with more than seven catalytic steps. The group of pathways larger than 7 steps included more complex variations of the shorter pathways and were discarded. The precursors of these vias were *E. coli *metabolites more distant from the central carbon and thus required the expression of more heterologous enzymes to be implemented in vivo than the shorter alternatives.

**Fig. 1:**
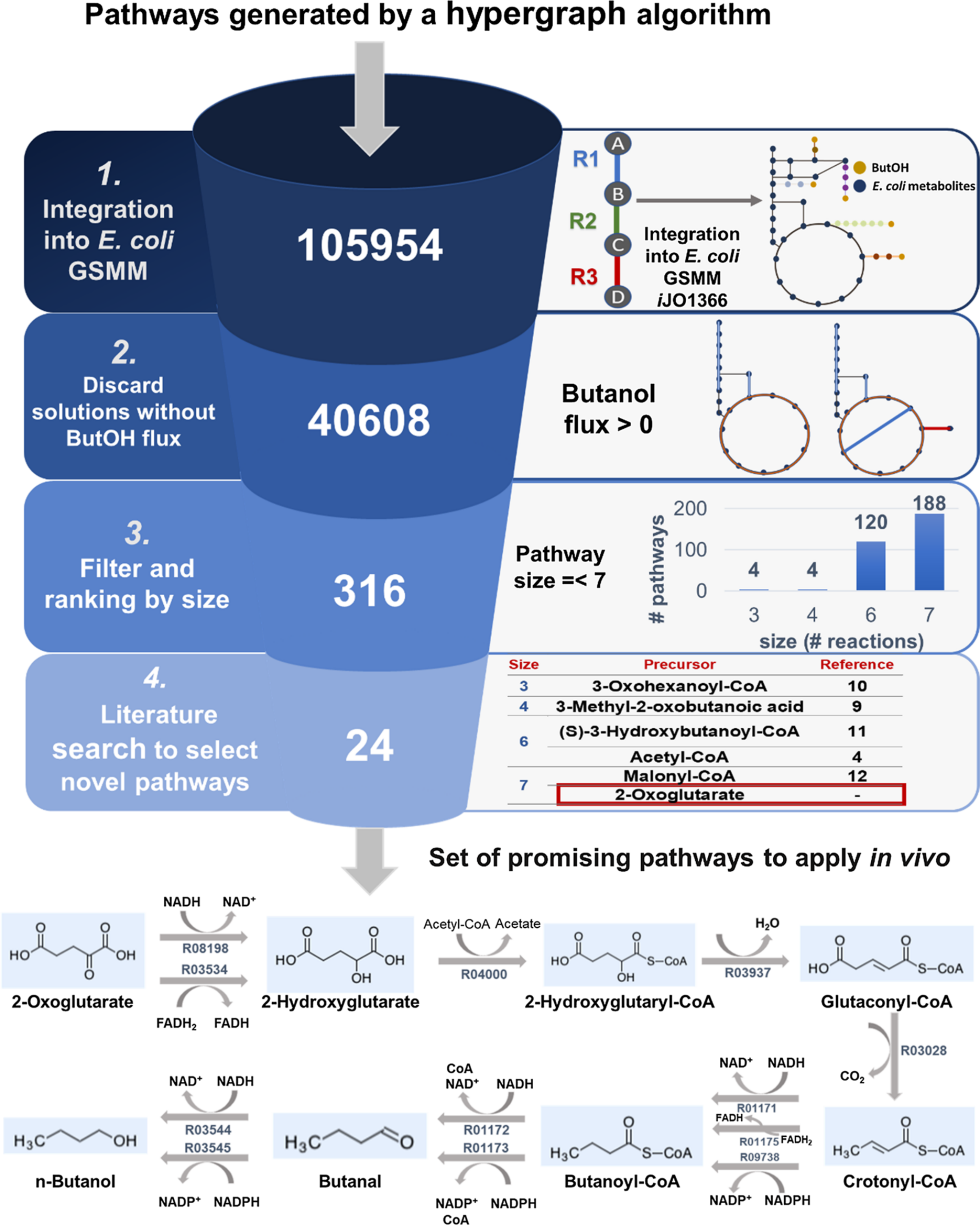
Schematic representation of the successive stages of the in silico analysis. The pathways to produce butanol in *E. coli* were generated using the algorithms described in [13], were filtered and ranked, resulting in a set of the 24 most promising alternatives. The reactions constituting the final set of pathways are shown with indication of the respective KEGG ID. These pathways are constituted by the following enzymatic activities: 2-oxoglutarate reductase (R08198 and R03534); glutaconate-CoA transferase (R04000); 2-hydroxyglutaryl-CoA dehydratase (R03937); glutaconyl-CoA decarboxylase (R03028); butyryl-CoA dehydrogenase (R01171; R01175 and R09738); aldehyde dehydrogenase (R01172 and R01173) and alcohol dehydrogenase (R03544 and R03545). *GSMM* genome-scale metabolic model, *ButOH* butanol

After reviewing the literature to infer their novelty, 24 pathways, all constituted by seven catalytic steps, have remained and considered the most auspicious for further analysis and in vivo implementation. Figure 1 depicts the various stages of the in silico analysis from the initial set of pathways, as well as a representation of the final set of pathways using 2-oxoglutarate as the main precursor.

In this set of pathways, first 2-oxoglutarate is reduced to 2-hydroxyglutarate via reaction R08198 or R03534 (all reaction identifiers are from KEGG [14–16]). Then, in reaction R04000, Coenzyme A is transferred from acetyl-CoA (releasing acetate) to 2-hydroxyglutarate forming 2-hydroxyglutaryl-CoA, followed by its dehydration into glutaconyl-CoA (reaction R03937). In the next reaction (R03028), glutaconyl-CoA is decarboxylated into crotonyl-CoA. The remaining reactions are common to clostridial pathway. Three different alternatives (R01171; R01175 and R09738) have been enumerated for the reduction of crotonyl-CoA in butanoyl-CoA. The last two steps are the successive reductions of butanoyl-CoA to butanal (R01173 or R01172) and of butanal to *n*-butanol (R03544 and R03545).

Although the main intermediary metabolites are common to all pathways present in the final set, the number of alternative routes [24] is a consequence of four out of the seven enzymatic steps allowing the use of different cofactors. The main novelty of this set of 24 pathways concerns the use of 2-oxoglutarate as the main precursor. 2-Oxoglutarate is a keto-acid that is an intermediate in TCA cycle and can be formed from the oxidation of isocitrate and subsequent decarboxylation of the intermediate oxalosuccinate. In anaerobic conditions, producing butanol from 2-oxoglutarate has a maximum theoretical yield similar to the clostridial and ketoacids pathways (clostridial and ketoacids: 0.41 g g^−1^; 2OG: 0.40 g g^−1^). The main difference between the two pathways is the amount of ATP that is produced in fermentative conditions with butanol as main product. Producing butanol using the 2OG pathway requires the carboxylation of one phosphoenolpyruvate molecule to oxaloacetate and the activation of 2-hydroxyglutarate to 2-hydroxyglutaryl-CoA using acetyl-CoA as a donor. These steps require extra energy input in comparison to the clostridial pathway, which makes the 2OG pathway less advantageous in this regard. Although the amount of ATP generated is lower, the 2OG pathway compensates in terms of thermodynamic feasibility (see “Pathway feasibility evaluation”). To the best of our knowledge, this set of pathways has not been reported in the literature and, for this reason, was selected for further analysis.

Some of the catalytic steps constituting the proposed pathway can be catalyzed using alternative cofactors (NADH or NADPH/FADH_2_). To infer what is the best combination of cofactors to produce butanol in *E. coli*, all reactions were added to the *E. coli* genome-scale metabolic model (GSMM) *i*JO1366, under anaerobic conditions, with glucose uptake rate equal to 10 mmol (g_DW_ h)^−1^. Butanol flux was then maximized to infer which reactions were active. As expected, in conditions favoring an excess of NADH, all the reactions recycling NADH had flux through butanol production and the remaining ones were inactive (Additional file 1: Table S1).

Thus, the pathway selected for further analysis utilizes only NADH as the donor of reducing power and was named pathway 2OG.

### Pathway feasibility evaluation

Since the KEGG database lacks information regarding directionality, all reactions are reversible by default. Therefore, it was necessary to check if each reaction in 2OG pathway occurs in the desired direction towards butanol; or if it is more likely to happen in the opposite way. The change in Gibbs Energy (∆_*r*_*G*) is a practical indicator for reaction directionality: a reaction will be favorable in a certain direction if ∆_*r*_*G* is negative, meaning Gibbs energy decreases during a reaction as ruled by the second law of thermodynamics [17]. For this reason, we calculated ∆_*r*_*G*′^*m*^, the change in Gibbs energy, considering reactants concentrations of 1 mM, a realistic concentration range at the cell context [18] using eQuilibrator [17]. Furthermore, to implement this pathway in vivo, we searched for possible genes coding for each of the required enzymatic activities. For each reaction active during the computational simulation (Additional file 1: Table S1), the estimated ∆_*r*_*G*′^*m*^ and respective gene sequences are shown in Table 1.

**Table 1:** Estimated change in Gibbs energy and experimental validation for the reactions constituting the 2-oxoglutarate pathway

Step	Reaction	Δ_*r*_*G*′^*m*^ (KJ mol^−1^)	*K*′_eq_	E.C. number	Enzyme	Gene NCBI accession no	Enzymatic activity^a^	Exp. validation in *E. coli*
1	*2-Oxoglutarate* + NADH + H^+^⟺ 2-Hydroxyglutarate^b^ + NAD^+^	− 22.6 ± 3.6	9.2 × 10^3^	EC 1.1.1.399	2-Oxoglutarate reductase	*hgdH* [AF] WP_012938338	[19]	[20, 21]
2	Acetyl-CoA + *2-Hydroxyglutarate* ⟺ Acetate + 2-Hydroxyglutaryl-CoA	− 8.6 ± 15.3	32.1	EC 2.8.3.12	Glutaconate CoA-transferase (subunits α and β)	*gctA* [AF] WP_012939156.1	[22]	
						*gctB* [AF] WP_012939155.1		
3	*2-Hydroxyglutaryl*-CoA ⟺ Glutaconyl-CoA + H_2_O	0.7 ± 2.9	0.751	EC 4.2.1.167	(R)-2-Hydroxyglutaryl-CoA dehydratase Subunits α, β and activator	*hgdA* [CS] WP_003501726.1	[23]	
						*hgdB* [CS] WP_003501727.1		
						*hgdC* [AF] WP_012939153.1	[24]	
4	*Glutaconyl-CoA* ⟺ Crotonyl-CoA + CO_2_	− 35.8 ± 17.4	1.9 × 10^3^	EC 1.3.8.6	Glutaryl-CoA dehydrogenase	*gcdH* [PA] WP_042912665.1	[25]	–
				EC 4.1.1.70	Glutaconyl-CoA decarboxylase α subunit	*gcdA* [CS] WP_003501720.1	[26]	–
5	*Crotonyl-CoA* + NADH + H^+^ ⟺ Butanoyl-CoA + NAD^+^	− 40.0 ± 17.0	1.0 × 10^7^	EC 1.3.1.44	*Trans*-2-enoyl-CoA reductase	*ter* [TD] WP_002681770.1	[27]	[5, 28–30]
6	*Butanoyl-CoA* + NADH + H^+^⟺ Butanal + CoA + NAD^+^	− 57.0 ± 16	9.6 × 10^9^	EC 1.2.1.57	Aldehyde/alcohol dehydrogenase	*adhE*1 [CA] WP_077851567.1	[31]	[32, 33]
7	*Butanal* + NADH + H^+^ ⟺ Butanol + NAD^+^	− 24.2 ± 4.8	1.8 × 10^4^	EC 1.1.1.1				

The estimated change in Gibbs free energy (Δ_*r*_*G*′^*m*^) values were computed with eQuilibrator [17] for each reaction constituting the novel pathway to produce butanol from 2-oxoglutarate (assumptions: reactant/product concentrations of 1 mM), as well as equilibrium constant (*K*′_eq_) values at a pH of 7 and an ionic strength of 0.1 M. The encoding gene and respective microorganism, as well as references of enzymatic activity validation with the substrate and experimental validation in *Escherichia coli* are also shown, plus the EC number and NCBI accession number

*Exp* experimental, *EC* enzyme commission, *AF Acidaminococcus fermentans*, *CS Clostridium symbiosum*, *PA Pseudomonas aeruginosa*, *TD Treponema denticola*, *CA Clostridium acetobutylicum*

^a^This refers to the substrate in italics in the reaction column

^b^Only the enzymes converting the (R)-isomer were considered. There are two enantiomers of 2-hydroxyglutarate; however, in KEGG (the database from which the reactions were retrieved), these variations are not represented. Depending on the enzyme, one of these forms can be preferably synthesized. The two subsequent reactions are part of the glutamate degradation pathway, existent is some microorganisms, where the (R)-isomers of the other compounds are preferably consumed

As shown in Table 1, the estimated ∆_*r*_*G*′^*m*^ values were negative for all reactions with exception of the third step (R03937), the dehydration of 2-hydroxyglutaryl-CoA into glutaconyl-CoA. In this case, the estimated ∆_*r*_*G*′^*m*^ value was close to 0 (0.7 ± 2.9 kJ mol^−1^), indicating that probably the reaction is reversible. Although a value of ∆_*r*_*G*′^*m*^ close to zero suggests that neither direction is favored, in physiological conditions, the flux can still be forced in the forward direction by increasing the concentration of 2-hydroxyglutatyl-CoA sufficiently above the concentration of the products [18]. The subsequent step (R03028) is much more favored in the forward direction with a ∆_*r*_*G*′^*m*^ of − 35.8 ± 17.4 kJ mol^−1^ and *K*′_eq_ 1.9 × 10^3^. So, we expect that this reaction will ensure the depletion of glutaconyl-CoA and drive the flux of the previous reaction in the desired direction. Regarding the other reactions of this pathway, the negative values of ∆_*r*_*G*′^*m*^ suggest that the production of butanol from 2-oxoglutarate is thermodynamically favorable.

After analyzing the literature, it was possible to conclude that all the catalytic steps constituting the proposed pathway have gene sequences available and experimental validation for the substrate. The first four reactions of the proposed pathway in Fig. 1 are part of the glutamate fermentation via the (R)-2-hydroxyglutarate pathway. This pathway, firstly described in 1974 [34], is present in different bacteria; the best known is the strictly anaerobic gut bacterium *Acidaminococcus fermentans* [34]. Moreover, the gene sequences selected for steps 1–3 were already validated for glutaconate production in *E. coli* [20].

The last steps of the pathway from crotonyl-CoA to butanol are common to clostridial pathway. For this reason, different enzymes catalyzing these reactions have been tested in *E. coli*. Regarding the reduction of crotonyl-CoA into butyryl-CoA, the expression of the clostridial butyryl-CoA dehydrogenase (Bcd-EtfAB) in *E. coli* is challenging due to its oxygen sensitivity and the requirement of ferredoxin as the electron donor [5, 35]. It was shown that another class of enzymes (*trans*-enoyl-reductases) also catalyzes this reaction increasing butanol titers when replacing the complex *bcd*-*etfAB* [5]. Thus, the protein product of *ter* from *Treponema denticola* was selected.

The two successive reductions of butyryl-CoA into butyraldehyde and to butanol are catalyzed by the bifunctional aldehyde/alcohol dehydrogenase. For these two last steps, the gene *adhE1* was selected since it was already validated for butanol production [32].

The main unknown regarding the novel butanol production pathway depicted in Fig. 1 concerns the decarboxylation of glutaconyl-CoA into crotonyl-CoA. This step is of increased importance because it is at the intersection between the upstream reactions—already experimentally validated for the production of glutaconate in *E. coli* [20]—and the downstream reactions, validated for the production of butanol [5] as shown in Table 1. Two genes were found to encode enzymes catalyzing this enzymatic step, but neither gave us enough confidence to apply directly in vivo because their activity was not previously validated in *E. coli*. The decarboxylation of glutaconyl-CoA into crotonyl-CoA can be catalyzed directly by glutaconyl-CoA decarboxylase [26], or, as an intermediate step, by glutaryl-CoA dehydrogenase [25]. For this reason, two different constructions were designed to test these two different enzymes for this particular step.

Glutaryl-CoA dehydrogenase is a bifunctional enzyme that catalyzes the decarboxylation of glutaconyl-CoA into crotonyl-CoA, using glutaryl-CoA as the initial substrate and glutaconyl-CoA as an intermediate [36]. Enzymatic assays showed that this enzyme was able to catalyze directly the conversion of glutaconyl-CoA into crotonyl-CoA with an activity 1.2–1.6 times higher than for the first step [25]. This enzyme was identified in cell-free extracts of *Pseudomonas* sp when grown anaerobically in aromatic compounds [25]. A curated gene sequence encoding this enzyme was available for *Pseudomonas aeruginosa* PAO1.

Glutaconyl-CoA decarboxylase is a four-subunit membrane-bound enzyme that catalyzes the conversion of glutaconyl-CoA into crotonyl-CoA, working as a biotin-dependent sodium pump [37]. This enzyme is involved in the fermentation of glutamate by strictly anaerobic bacteria. Glutaconyl-CoA decarboxylases from the bacteria *Acidaminococcus fermentans* [38] and *Clostridium symbiosum* [26] were crystalized and characterized. Considering that the butanol production pathway tested here requires a high number of heterologous genes, we decided to express only the α-subunit of the glutaconyl-CoA decarboxylase (encoded by *gcdA*), which is able to catalyze the reaction as long as biotin is also supplied. Regarding the microorganism source, within the best studied glutaconyl-CoA decarboxylases, we have selected *Clostridium symbiosum* because the *K*_*m*_ for biotin was 14-times lower than the value obtained for the α-subunit of glutaconyl-CoA decarboxylase from *A. fermentans* [26].

### In vivo implementation

**Fig. 2:**
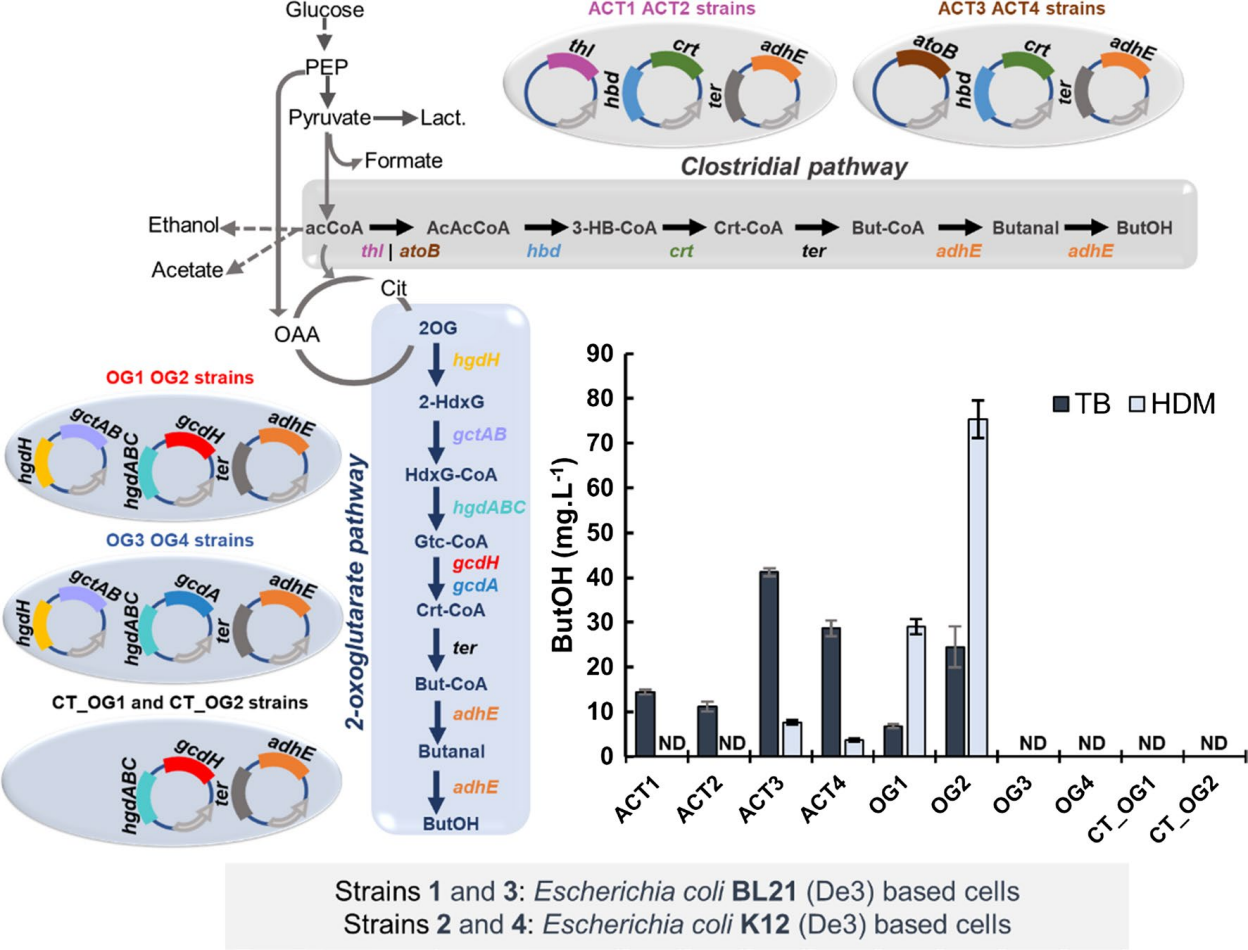
Butanol titer (mg L^−1^) for the different strain designs in TB medium and HDM medium. The different enzymes expressed in each strain are indicated. In all experiments, strains were grown in shake flasks until reaching 0.4–0.5 OD_600_. 0.5 mM of IPTG was then added to the medium and cells transferred to sealed serum bottles. Data are shown as mean ± SD from three independent experiments. *ND* not detected; the detection limit of the method is 3 mg L^−1^. *2OG* 2-oxoglutarate, *2-HdxG* 2-hydroxyglutarate, *HdxG-CoA* 2-hydroxyglutaryl-CoA, *Gtc-CoA* glutaconyl-CoA, *Crt-CoA* crotonyl-CoA, *But-CoA* butanoyl-CoA, *ButOH* butanol, *acCoA* ACETYL-CoA, *AcAcCoA* acetoacetyl-CoA, *3-HB-CoA* 3-hydroxybutyryl-CoA, *Cit* citrate, *Lact* lactate, *OAA* oxaloacetate, *PEP* phosphoenolpyruvate

Two different constructions were designed with all the genes constituting the pathway and two different enzymes for the fourth step: one expressing a glutaryl-CoA dehydrogenase (*gcdH*) from *Pseudomonas aeruginosa* and another one expressing the α-subunit of glutaconyl-CoA decarboxylase (*gcdA*) from *C. symbiosum*. These genes were introduced in BL21 (De3) (OG1 and OG3)- and K-12-based cells (OG2 and OG4) as depicted in Fig. 2. The developed strains were cultivated in the complex medium TB and in the synthetic HDM medium, induced with 0.5 of IPTG and immediately switched to sealed serum bottles. The results are summarized in Fig. 2.

The butanol titers obtained by testing the fours strains expressing the 2OG pathway showed that this compound could only be detected in strains where glutaryl-CoA dehydrogenase (OG1 and OG2) was expressed (detection limit is 3 mg L^−1^). Data retrieved from the literature showed that the sole expression of the α-subunit of glutaconyl-CoA decarboxylase has a *V*_max_ 1000 times lower than the full enzyme [26]. Therefore, it is not possible to affirm that this enzyme is not a viable option for the proposed pathway. Moreover, there is a discrepancy in the copy number of the plasmid used for expressing the two enzymes: pCOLADuet possesses 20–40 copy number per cell (plasmid used for *gcdA*), while pRSFDuet (the plasmid used to express *gcdH*) has a copy number higher than 100 plasmids per cells. To confirm that *gcdH* is more suitable for this pathway, both genes should be cloned using the same conditions. Given the lack of butanol production in the strains expressing *gcdA* and the challenge of expressing all four subunits of the transmembrane glutaconyl-CoA decarboxylase, further experiments with this pathway were performed only for the strains using the glutaryl-CoA dehydrogenase.

As depicted in Fig. 2, we implemented a few variations of the clostridial pathway in *E. coli* and compared with the 2OG pathway in terms of butanol production using the same culture conditions. For the first step of this route—the thermodynamically unfavorable condensation of two molecules of acetyl-CoA into one of acetoacetyl-CoA—two enzymes were tested: the protein products of the clostridial *thl* and of the *E. coli* native *atoB*. Both derivative pathways were expressed in BL21 (ACT1 and ACT3) and in K12 cells (ACT2 and ACT4). Butanol accumulation was detected in both designs either expressing *thl* or *atoB*. In accordance to the data previously reported, the cells expressing *atoB* (ACT3 and ACT4) achieved higher butanol titers than the ones expressing *thl* (ACT1 and ACT2) [9].

The maximum butanol titer obtained in serum bottles (75 ± 4 mg L^−1^) was achieved by cultivating OG2 (*E. coli* K12 host) in HDM medium. This value is 1.8-fold greater than the maximum butanol production obtained for the ACT strains (41 ± 8 mg L^−1^), presenting a good indicator of the potential of this novel pathway.

For both OG1 and OG2 strains, HDM formulation seems to favor butanol production over TB medium. Contrarily, strains expressing clostridial pathway (ACT) achieved higher butanol titers in TB medium. Other publications have demonstrated that the presence in the medium of complex nutrients is beneficial for butanol production through the clostridial pathway and its derivatives. By removing complex nutrients such as tryptone and yeast extract from the medium, the authors observed a reduction in butanol production [5, 30]. The contrasting behavior between the two butanol production pathways is likely to be a consequence of the different precursors used by each pathway and the influence of the complex nutrients on their availability.

To determine if the medium formulation used here could be further optimized, we omitted some of the extra components added in the synthetic medium formulation (HDM) (Additional file 2: Fig. S1). The formulation that provided the highest butanol titer for OG2 was the one including all the supplements. Surprisingly, for OG1, the omission of iron (III) citrate and riboflavin had a positive impact in the final butanol titer, representing a 1.5-fold improvement when compared with the complete formulation of HDM. Iron(III) citrate and riboflavin were supplemented due to the reported requirements of 2-hydroxyglutarate dehydratase (encoded by the genes *hgdABC*) [20]. Given the direct connection between butanol production in the strains tested and 2OG, we expected that the omission of glutamate would cause the greatest impact on butanol titer. However, the results actually showed that omitting the mixture of amino acids resulted in a lower butanol accumulation than the single omission of glutamate.

Regarding the host strain used for butanol production, we observed that BL21 strains provided the highest butanol titers when expressing clostridial pathway and K12 based strains showed increased butanol production through the 2OG pathway.

To validate that the pathway introduced into *E. coli* was using 2-oxoglutarate as starting precursor, strains expressing only a part of the pathway, CT_OG1 and CT_OG2, were cultured in the same fashion as described before for experiments using TB and HDM media. These strains only expressed the final five catalytic steps of the pathway. The main goal was to infer if some alternative precursors were being used directly from the media by the enzymes catalyzing the final five reactions of the proposed pathway. Since butanol was not detected in all the performed experiments, we can conclude that all the three plasmids are needed to produce a detectable amount of butanol.

### Butanol production in bioreactors

Under anaerobic conditions, *E. coli* produces mixed-acid fermentation products decreasing the pH of the medium, which can slow, or even stop growth. For this reason, OG2 (the strain that provided the greatest titers in the serum bottle experiments—Fig. 2) was cultivated under controlled conditions in a bioreactor.

**Fig. 3:**
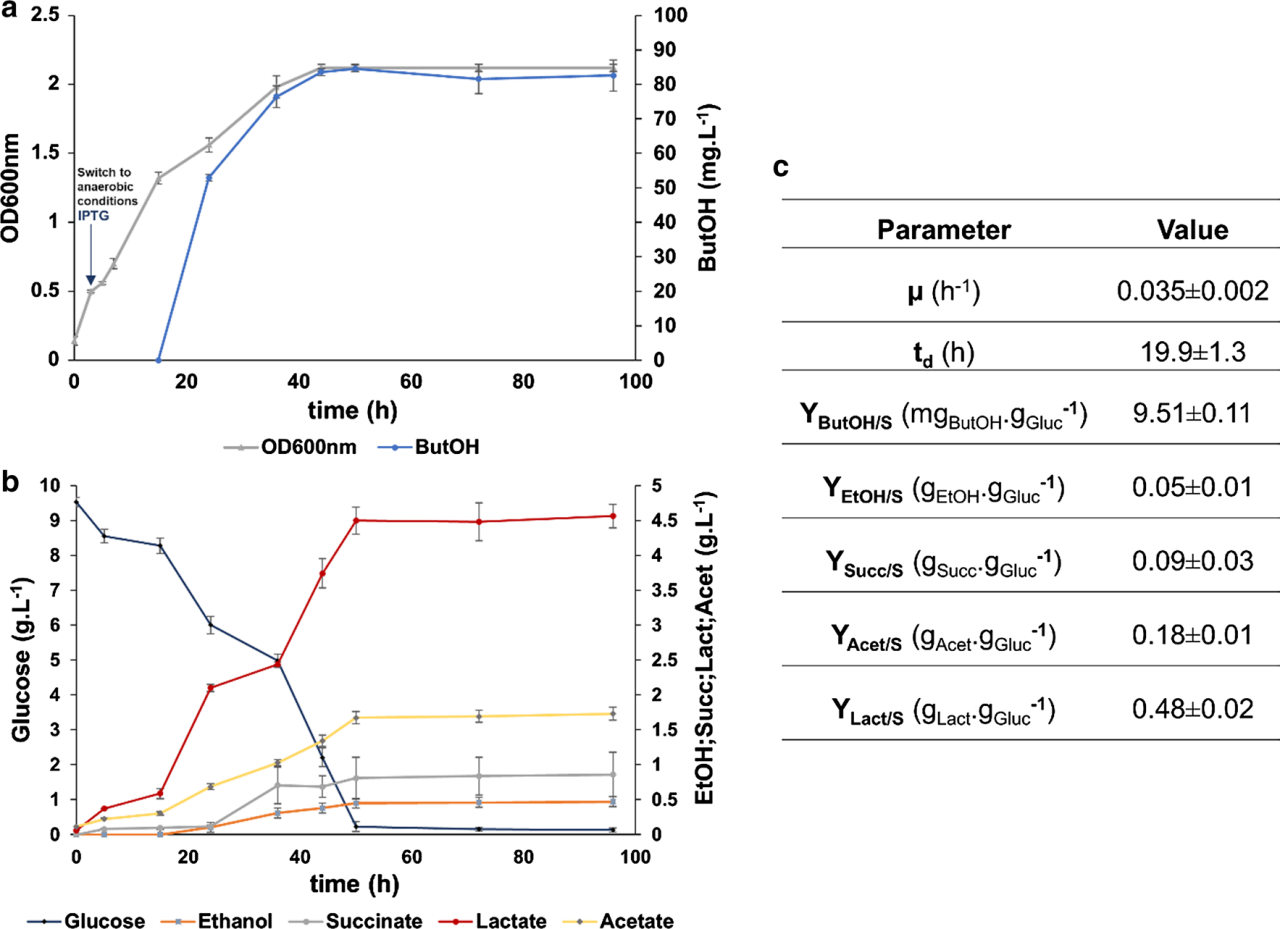
Physiological characterization of the strain OG2 in bioreactor. **a** Growth-curve (optical density at 600 nm) profile and butanol concentration; **b** glucose and end-products concentrations during the fermentation and **c** specific growth rate (*µ*), duplication time (*t*_d_) and butanol, ethanol, succinate, acetate and lactate yields (*Y*) on glucose. Cells were cultivated aerobically in HDM medium and induced with 0.5 mM of IPTG at 0.4–0.5 OD_600_. At this moment, anaerobic conditions were created by turning off the air flow and waiting for the leftover oxygen to be consumed. Data are shown as mean ± SD of three independent experiments. *ButOH* butanol, *EtOH* ethanol, *Succ* succinate, *Acet* acetate, *Lact* lactate

OG2 was cultivated with 0.5-l working volume in a 2-l bioreactor using HDM medium. Samples were taken to measure cell density and concentration of excreted metabolites with HPLC and GC-FID analysis. Figure 3 summarizes the obtained results.

It is possible to observe in Fig. 3 that the maximum butanol titer (85 ± 1 mg L^−1^), obtained after 50 h, represented a 1.1-fold improvement, comparing with the results obtained in serum bottles for OG2 strains (Fig. 2). We can see a biomass-product coupled behavior by analyzing the growth curve and the butanol production profile in Fig. 3a. During the first 3 h, there is growth under aerobic conditions and at this moment, 0.5 mM of IPTG was added to the medium. Between 5 and 12 h, the growth rate and glucose consumption decreased. The switch to anaerobic conditions and the metabolic burden imposed by IPTG induction can explain this deceleration. Next, between 12 and 50 h, cells continuously grew, consuming glucose, producing butanol and mixed-acid fermentation products until reaching a stationary phase.

Butanol was first detected at 24 h of fermentation and its accumulation increased until 50 h. For this moment on, butanol concentration remained constant, coinciding with the glucose exhaustion and biomass growth plateau. In the experiments using serum bottles (Fig. 2), the greatest titer was only achieved after 96 h of cell growth. So, we can conclude that cultivating the cells under controlled conditions has accelerated butanol production, although the increment on titers has been modest. Nevertheless, even in bioreactors, the growth rate (*µ*) was low (0.035 ± 0.002 h^−1^), representing a duplication time of 19.9 ± 1.3 h. Analyzing Fig. 3b, lactate was the major fermentation product released by the cells. In fact, the yield of lactate on glucose was 0.48 ± 0.02 *g*_Lac_ *g*_gluc_^−1^. Butanol yield on substrate was the lowest one within the detected products, namely succinate, ethanol, lactate and acetate. So, during the anaerobic growth, *E. coli* preferably uses the native pathways to recycle the excess of NADH instead of the butanol pathway, even with the high amount of heterologous proteins being presumably expressed. This was also observed by other groups expressing the clostridial pathway in *E. coli*, in which butanol accumulation increased after deleting mixed-acid fermentation pathways [5, 28].

## Discussion

The main objective of this study was to rationally design a new microbial cell factory able to produce butanol through a novel pathway, generated by a hypergraph algorithm. Furthermore, the analysis carried using several in silico tools aimed to save time in the lab by avoiding a trial and error experimental approach.

From the initial set of 105,954 different pathways, only 40,608 routes could actually produce butanol when integrated into an *E. coli* model. Since stoichiometry is not considered by the enumeration algorithm, the FBA simulations allowed to identify the stoichiometrically feasible pathways, i.e., with butanol flux. The successive filters applied (based on size and novelty) allowed the identification of a set of 24 pathways. These pathways catalyze, with some alternative reactions, the conversion of 2-oxoglutarate into *n*-butanol. Within *E. coli* and maximizing butanol production in silico under anaerobic conditions, NADH would be the most efficient cofactor to produce butanol using this pathway. By retrieving the literature and analyzing databases, we selected the most suitable enzymes for the different steps of this pathway. The in vivo implementation of this pathway allowed butanol production when the fourth step was catalyzed by glutaryl-CoA dehydrogenase (OG1 and OG2) expressed in pRSFDuet, but not when the α-subunit of the glutaconyl-CoA decarboxylase was used in its place (OG3 and OG4) using an expression vector with a lower copy number—pCOLADuet. To the best of our knowledge, this was the first time that butanol was produced in *E. coli* through this pathway using 2OG as precursor.

*Escherichia coli* K12 MG1655 was used as host for expressing the 2OG pathway, considering that all the in silico simulations were based on *i*JO1366 [39], a genome-scale metabolic model developed for this strain. We also tested *E. coli* BL21 (DE3) to produce butanol for being considered more suitable for heterologous gene expression. In fact, *E. coli* genome had been modified in BL21 (DE3) to improve protein expression, lacking genes encoding proteases (such as *Lon* and *OmpT*) and preventing plasmid loss by the mutation in gene *hsdB* [40]. Nevertheless, the results (Figs. 2, 3) showed that *E. coli* K12 produced higher titers of butanol through the 2OG pathway than the BL21 equivalent. Since recombinant protein production is expected to be favored in BL21 hosts, a possible explanation for the results may be the metabolism of the two different strains. Reportedly, in BL21 cells, the enzymes responsible for the glyoxylate bypass are constitutively expressed; while in K12 cells, their expression is extremely regulated [41]. This pathway converts directly isocitrate and acetyl-CoA into malate and succinate, avoiding the two successive decarboxylations of isocitrate into 2OG and succinyl-CoA [41]. Assuming that the glyoxylate bypass is only active in OG1 strains when glucose is the main carbon source, the respective flux through the oxidative branch of the TCA cycle will be lower than in OG2. Consequently, the accumulation of 2OG will be reduced [42]. So, we hypothesize that, in OG2 strains, the 2OG pool is higher, increasing the flux towards the heterologous production of butanol. Moreover, the superior butanol production results when expressing the clostridial pathway in BL21-based strains (Fig. 2) support this hypothesis, since acetyl-CoA is the main precursor of the clostridial pathway. As future work, the expression of 2OG pathway into *E. coli* K12 JM109 hosts could provide higher butanol titers, since the genome of these K12-based strains was modified to enhance recombinant protein expression, similarly to BL21 cells [43].

It is important to point out that the major difference between the two pathways is the precursor. Both metabolites (acetyl-CoA and 2-oxoglutarate) are part of the central carbon metabolism and intermediates in fundamental biochemical pathways. The immediate advantage of the 2OG pathway is the thermodynamically favorable first step (Δ_*r*_*G*′^*m*^ = − 22.6 ± 3.6 kJ mol^−1^) to avoid the unfavorable condensation of two molecules of acetyl-CoA (Δ_*r*_*G*′^*m*^ = 26.1 ± 1.7 kJ mol^−1^). However, under anaerobic conditions, the TCA cycle is downregulated and 2OG is only synthesized to support biomass formation. The impact of draining 2-oxoglutarate from the TCA cycle has not been studied before. In the literature, there are examples of successfully expressed heterologous pathways in *E. coli* using 2OG as substrate for the production of glutaconate [20] and glutarate [21] under anaerobic conditions. In these works, the titers achieved are not large; so, it is not possible to infer the effect of the 2OG depletion. The simulations we performed with the novel butanol pathway showed that even when all pathways for mixed-acid fermentation are deleted (lactate, ethanol, acetate) the 2OG pathway is enough to support anaerobic growth with a reduction of the maximum growth rate to 50% of the wild type.

OG strains accumulated more butanol in the defined medium than in the complex counterpart (Fig. 2). Hence, the production of butanol seems to be positively affected by the defined HDM formulation. We hypothesize that the supplementation in HDM medium of amino acids in the free form—tryptone could contain small peptides in TB medium—positively impacts the 2OG pool increasing butanol accumulation. This is also supported by the data shown in Additional file 2: Fig. S1, where the omission of amino acids solution from HDM medium caused the highest reduction on butanol titers. The opposite results obtained with ACT strains, where TB medium provided the maximum butanol titers, agree with what is reported in the literature, where the omission of complex nutrients had a negative impact on butanol accumulation when expressing the clostridial pathway [4, 5, 30, 44]. The hypotheses for this behavior include the high demand of this pathway for acetyl-CoA and NADH, limiting the available resources for protein synthesis [30] and the role of yeast extract as a nitrogen donor, increasing biomass growth and butanol titers [5].

The obtained titers are far below the industrial requirements, and thus further optimization to achieve industrial requirements is needed. Additional metabolic engineering strategies can help to develop more efficient cell factories. An obvious next step is the already mentioned deletion of the pathways for the competing fermentation products. Further exploration of strategies to improve the accumulation of the precursor 2-oxoglutarate could help to improve butanol titers obtained through the respective pathway, while the detection of pathway intermediates could allow to identify the bottleneck(s), providing insight for the next steps for strain engineering [28].

Other strategies include the use of RBS strength prediction algorithms or manipulation of mRNA stability [45] to fine-tune gene expression [46]. Also, application of techniques such as transcriptomics, fluxomics and metabolomics could give insight on the metabolism of the different strains, allowing to identify possible metabolic engineering strategies to increase butanol accumulation [47].

## Conclusions

In this work, we were able to design novel strains of *E. coli* capable of producing butanol through a novel pathway generated by a hypergraph algorithm using 2-oxoglutarate as the precursor.

*Escherichia coli* OG2, using *E. coli* K12 MG1655 DE3 as host, provided higher final titers of butanol than using BL21. The greatest titer (75 ± 4 mg L^−1^) was obtained by cultivating OG2 strains in HDM medium and inducing with 0.5 mM of IPTG.

The maximum titers obtained for this novel pathway are still far below from those required for industrial purposes. The results obtained in the bioreactor indicate that the cells expressing 2OG pathway preferably recycle NADH using native mixed-acid fermentation pathways instead of the heterologous pathway. The knock-out of mixed-acid fermentation pathways probably is necessary to force NADH recycling using the heterologous pathway. These metabolic engineering strategies were coupled with other approaches to increment the 2OG availability and are ongoing.

## Materials and methods

### In silico analysis of heterologous pathways

Several alternative routes to produce *n*-butanol were generated using an hypergraph algorithm previously developed within our group [13] based on FindPath (FP) [48] using a proprietary database as the search space. The aim was to enumerate all possible combinations of reactions leading from any reaction at the central carbon metabolism of *Escherichia coli* to *n*-butanol. The solutions obtained were analyzed to select the most promising ones to implement in vivo. The first step of this analytical process was to test if the pathways would allow butanol production when inserted in the *i*JO1366 *E. coli* GSMM [39]. This test was carried using FBA (flux balance analysis) with maximization of butanol flux as the objective function, for each pathway generated. Since stoichiometry is not considered by the enumeration algorithm, the FBA simulations without butanol production (i.e., maximum butanol flux = 0) allowed to identify the stoichiometrically infeasible pathways. Pathways without any butanol flux were discarded and diverse filters were successively applied to the remaining pathways. First, the pathways were sorted by size, i.e., the number of reactions needed to catalyze the initial precursor into butanol. Since clostridial pathway is constituted by six steps, the maximum threshold for pathway size was set to seven. Within the diverse groups sorted by size, the pathways were ranked accordingly to the conservation of number of carbon atoms (i.e., the difference between the number of carbons of the initial substrate and butanol). For the pathways with the greatest conservation of carbon atoms, a manual analysis was carried out.

To assure the novelty of the chosen pathways, the relevant literature was searched for any previous reports of butanol production using the same set of reactions and the ones already reported were discarded. Then, the availability of curated gene sequences encoding the enzymes that catalyze the different reactions was verified by consulting databases such as UniProt [49], KEGG [14–16], and MetaCyc [50]. For all the reactions constituting the most promising set of pathways, the change in Gibbs free energy (Δ_*r*_*G*′^*m*^) and Equilibrium constant (*K*′_eq_) were calculated using eQuilibrator 2.0 [17] to evaluate their reversibility. These values were estimated using Component Contribution [51] considering reactants concentrations of 1 mM, a pH of 7 and an ionic strength of 0.1 M.

### Genome-scale model and software

The reactions constituting the most promising pathway obtained from the analysis described in the previous section were again added to *i*JO1366 *E. coli* GSMM [39]. Additional file 3: Table S2 shows the stoichiometry of the reactions added to the model, as well as transport reaction to allow the excretion of butanol. Reaction R01175 was not added to the GSMM, since the model already includes this reaction (R_ACOAD1f). The reaction (*S*)-3-hydroxybutanoyl-CoA hydrolyase (R_ECOAH1) was deleted from the GSMM to make sure that butanol production originated from the heterologous pathway.

*OptFlux*3 [52] and FBA were used to perform all in silico analyses. *n*-Butanol production was studied under anaerobic (oxygen uptake flux was set to 0 mmol (g_DW_ h)^−1^) environmental conditions, with the glucose uptake rate set to 10 mmol (g_DW_ h)^−1^ and ammonia, phosphate and sulfate uptake unconstrained and using butanol export reaction as the objective function. Flux variability analysis (FVA) allowed to infer the robustness of butanol production, i.e., to determine the flux limits of the active reactions under anaerobic conditions.

### Strains and plasmids

The strains and plasmids used or constructed in this study as well as all the primers used are listed in Additional file 4: Tables S3, S4 and S5.

### Cloning procedure

Basic molecular biology techniques were employed as previously described [53]. The genes used in this study were amplified by polymerase chain reaction (PCR) using Phusion High-Fidelity DNA Polymerase (Thermo Scientific, Waltham, USA) in a LifeECO Thermal Cycler (Bioer Technology, Zhejiang, China). All primers were purchased from Metabion (Munich, Germany). DNA fragments were purified using DNA Clean and Concentrator DNA Kit (Zymo Research, Irvine, USA).

Plasmids were extracted using Plasmid Miniprep kit (Zymo Research). All digestions were performed using the appropriate FastDigest^®^ restriction endonucleases (Thermo Scientific). Ligations were performed with T4 DNA Ligase (Thermo Scientific) and transformed by heat-shock in chemically competent *E. coli* NEB 5-alpha cells (New England BioLabs, Massachusetts, USA). The success of ligation was checked through Colony PCR using DreamTaq (Thermo Scientific) and further confirmed by sequencing (StabVida, Lisbon, Portugal). Protocols were performed in accordance with manufacturer’s instructions.

### Shake flasks and sealed flasks experiments

Butanol production experiments were performed in Terrific Broth (TB) medium [54] and high-density medium (HDM) adapted from [55].

TB medium contained, per liter, tryptone (12 g); yeast extract (24 g); glycerol (4 mL); monobasic potassium phosphate (2.31 g) and dibasic potassium phosphate (12.54 g). The pH of this medium was 7.2 ± 0.2 at 25 °C; adjustments were not necessary.

HDM formulation contained (per liter) dibasic sodium phosphate dihydrate (8.89 g); monobasic potassium phosphate (6.8 g); sodium chloride (0.58 g); magnesium sulphate (1.35 g); calcium chloride dihydrate (0.038 g); ammonium chloride (1 g); trace metals (250 µL); vitamins BME 100× (250 µL) and an amino acid mix (2 g).

The trace metals solution contained (per liter): FeSO_4_·7H_2_O (30 mg); ZnSO_4_·7H_2_O (45 mg); CaCl_2_·2H_2_O (45 mg); MnCl_2_·2H_2_O (100 mg); CoCl_2_·6H_2_O (30 mg); CuSO_4_·5H_2_O (30 mg); Na_2_MoO_4_·2H_2_O (40 mg); H_3_BO_3_ (10 mg); KI (10 mg) and Na_2_EDTA (1.5 g). The amino acid mix contained 1 g of adenine and 4 g of arginine, aspartate, glutamate, histidine, isoleucine, lysine, methionine, phenylalanine, serine, threonine, tryptophan, tyrosine and valine. The vitamin BME 100× solution (Sigma Aldrich, St. Louis, MO, USA) contained (per liter): d-biotin (0.1 g); choline chloride (0.1 g); folic acid (0.1 g); myo-inositol (0.2 g); niacinamide (0.1 g); D-pantothenic acid. ½Ca (0.1 g); riboflavin (0.01 g); thiamine·HCl (0.1 g) and NaCl (8.5 g). The pH of the medium was adjusted to 7.1 at 25 °C using 2 M NaOH.

For strains expressing 2OG pathway, both media were supplemented, per liter, with 10 g glucose; 0.468 g glutamate; 0.0753 g riboflavin and 0.525 g iron (III) citrate.

Luria–Bertani (LB) medium contained 10 g L^−1^ of peptone; 5 g L^−1^ yeast extract and 5 g L^−1^ of NaCl. The solid version of this medium included 15 g L^−1^ agar.

All cultivations were performed with the addition of suitable antibiotics according to the employed plasmids. For OG and ACT strains, the antibiotics concentrations were 50 µg mL^−1^ ampicillin, 50 µg mL^−1^ spectinomycin, and 30 µg mL^−1^ kanamycin. For control strains, the concentrations employed were 50 µg mL^−1^ ampicillin and 30 µg mL^−1^ kanamycin.

A single colony was picked from LB plates and inoculated in 10 mL of liquid LB medium. The pre-cultures were grown aerobically on a rotary shaker at 37 °C and 200 rpm, overnight.

In HDM experiments, an appropriate volume of cells was harvested from the pre-culture by centrifugation (10 min at 3000×*ɡ*) and washed with HDM medium and then transferred to 500-mL shake flasks with 100 mL of medium, yielding an initial OD_600_ of 0.1. In TB experiments, an appropriate volume of pre-culture was directly transferred to 500-mL shake flasks with 100 mL of medium, yielding an initial OD_600_ of 0.1. These cultures were also cultivated on a rotary shaker at 200 rpm at 37 °C. Butanol production genes were induced with 0.5 mM IPTG at an OD_600_ of 0.4–0.5.

To promote butanol production, after induction, the cells were switched to anaerobic conditions by transferring 60 mL of culture to 120-mL sealed serum flasks. The culture was supplemented with 600 µL of a 0.01 M stock solution of sodium bicarbonate to achieve a final concentration of 10 mM, since it reduces long lag phases in *E. coli* anaerobic growth [56]. 60 µL of a solution of extra trace metals [NiCl_2_ (1.7 mg L^−1^); (NH_4_)_6_Mo_7_O_24_ (14.5 mg L^−1^); 4H_2_O Na_2_SeO_3_ (2.4 mg L^−1^)] was supplied to the medium since selenium, nickel and molybdenum are part of the formate hydrogen lyase (FHL) complex, which is induced under anaerobic conditions [57]. After induction, the cultures were incubated at 30 °C and 180 rpm, for 96 h.

Samples of broth were collected at time 0, induction time and 96 h for OD_600_ measurements and high-performance liquid chromatography (HPLC) and gas chromatography–flame ionization detector (GC–FID) analysis of the supernatant. All experiments were performed in triplicate.

### Bioreactor cultivations

Bioreactor fermentations were performed in HDM medium. Cells were pre-grown overnight in 500-mL shake flasks containing 100 mL of the same medium, at 37 °C and 200 rpm. Each fermenter was inoculated at an initial OD_600_ of 0.15. The fermentations were performed in the Eppendorf DASGIP Parallel Bioreactor System (Switzerland) using 2-L culture vessels. The operating volume for the fermentations was 0.5 L, temperature was maintained at 37 °C, airflow at 1 VVM, pH was kept at 7.0 controlled by addition of 2 M NaOH, and dissolved oxygen was kept above 30% of saturation by feedback control of the stirring speed from 200 rpm up to 400 rpm. Expression of the butanol genes was induced with 0.5 mM of IPTG when an OD_600_ of 0.4–0.5 was reached. After IPTG induction, temperature was decreased to 30 °C and stirring speed to 180 rpm. Anaerobic conditions were created by turning off the air flow and waiting for the leftover oxygen to be consumed. Samples were taken every 2 h for the first 12 h of the fermentation and, then, every 12 h. At each time point, the optical density was measured, and the supernatant was analyzed by HPLC and GC–FID.

### Analytical methods

Butanol was quantified by GC and organic acids, ethanol and glucose by HPLC.

Samples were centrifuged at 6000×*ɡ* for 10 min to separate cells from the medium. Afterwards, the supernatant was filtered with a 0.22 µm pore filter membrane to glass vials and stored at − 20 °C until analyzed.

Quantitative analysis of organic acids and glucose was performed using a HPLC apparatus from Jasco (Japan) model LC-NetII/ADC equipped with UV-2075 Plus and RI-4030 Plus detectors, also from Jasco. The samples were analyzed using an Aminex HPX-87H column (300 mm × 7.7 mm) from Bio-Rad, which was kept at 60 °C, and 5 mmol L^−1^ H_2_SO_4_ was used as mobile phase with a flow rate of 0.5 mL min^−1^. Glucose and ethanol were detected with the refractive index (RI) detector and organic acids (succinate, lactate, formate and acetate) were detected at 210 nm using the UV detector. Calibration curves were obtained by injecting standards with known concentrations for each metabolite. Metabolite concentrations in samples were calculated by comparing the peak areas of the samples with the calibration curves.

Butanol concentration was quantified by a GP-9000 system (Chrompack) with a Meta-WAX capillary column (30 m × 0.25 mm × 0.25 µm) equipped with a flame ionization detector (FID) where Helium was used as carrier gas with a flow rate of 1 mL min^−1^. The filtered supernatant (900 µL) was mixed with 100 µL of a 5 g L^−1^ solution of isobutanol, the internal standard, yielding a final concentration of 0.5 g L^−1^, and 1 µL of this mixture was injected. The temperatures of injector and detector were maintained at 250 °C. The column temperature was initially at 50 °C, heated to 177.5º C at a 5 °C min^−1^ rate and then heated to 230 °C at 10 °C min^−1^, which was held for 15 min. A calibration curve was obtained by injecting standards with several concentrations of butanol and a fixed concentration of internal standard (0.5 g L^−1^ of isobutanol). Butanol concentration was calculated by comparing the ratio between its peak area and internal standard peak area with calibration curves.

All optical density measurements at 600 nm (OD_600_) were performed using the spectrophotometer Ultrospec 10 from Biochrom (Cambridge, UK).

## Supplementary information

**Additional file 1: Table S1.** Simulation results using flux balance analysis and flux variability analysis.

**Additional file 2: Fig. S1.** Medium formulation influence on butanol accumulation.

**Additional file 3: Table S2.** Reactions added to *Escherichia coli* genome scale metabolic model *i*JO1366.

**Additional file 4: Table S3.** Sequences of primers used in the cloning procedures of this study; **Table S4.** List of plasmids used or designed in this study. **Table S5.** List of strains and genomic DNA used or engineered for this study.

## Data Availability

The datasets used and/or analyzed during the current study are available from the corresponding author on reasonable request.

## Abbreviations

2OG: 2-oxoglutarate
2-HdxG: 2-hydroxyglutarate
HdxG-CoA: 2-hydroxyglutaryl-CoA
3-HB-CoA: 3-hydroxybutyryl-CoA
ABE: acetone–butanol–ethanol
AcAcCoA: acetoacetyl-CoA
acCoA: acetyl-CoA
Acet: acetate
AF: *Acidaminococcus fermentans*
But-CoA: butanoyl-CoA
ButOH: butanol
CA: *Clostridium acetobutylicum*
Cit: citrate
Crt-CoA: crotonyl-CoA
CS: *Clostridium symbiosum*
EC: enzyme commission
EtOH: ethanol
Exp: experimental
FBA: flux balance analysis
FID: flame ionization detector
FP: FindPath
FVA: flux variability analysis
GC: gas chromatography
GSMM: genome-scale metabolic model
Gtc-CoA: glutaconyl-CoA
HDM: high-density medium
HPLC: high-performance liquid chromatography
IPTG: isopropyl β-1-thiogalactopyranoside
KEGG: Kyoto Encyclopedia of Genes and Genomes
Lact: lactate
LB: Luria Bertani
ME: metabolic engineering
OAA: oxaloacetate
OD_600_: optical density at *λ*_600_ nm
PA: *Pseudomonas aeruginosa*
PCR: polymerase chain reaction
PEP: phosphoenolpyruvate
RI: refractive index
Succ: succinate
TCA: tri-carboxylic acid
TB: Terrific Broth
TD: *Treponema denticola*
UV: ultraviolet
