# Supplementary material for: Discovery and implementation of a novel pathway for n-butanol production via 2-oxoglutarate
Source: Biotechnol Biofuels. 2019 Sep 30;12:230. doi: 10.1186/s13068-019-1565-x (PMC6767645; doi:10.1186/s13068-019-1565-x)
Supplement: Supplementary file 1 — Additional file 1: Table S1. Simulation results using flux balance analysis and flux variability analysis. [file 13068_2019_1565_MOESM1_ESM.docx]

**Additional file 1: Cofactor usage analysis**

**Table S1 Simulation results using Flux Balance Analysis and Flux Variability Analysis.**

| Catalytic step | Reaction | KEGG ID | Flux (mmol.(g_DW_.h)^-1^) |
| --- | --- | --- | --- |
| 1 | 2-Oxoglutarate + **FADH_2_** <=>2-Hydroxyglutarate + **FAD** | R03534 | - |
|  | 2-Oxoglutarate + **NADH** + H^+^ <=> 2-Hydroxyglutarate + **NAD^+^** | **R08198** | **8.26** |
| 2 | Acetyl-CoA + 2-Hydroxyglutarate <=> Acetate + 2-Hydroxyglutaryl-CoA | **R04000** | **8.26** |
| 3 | 2-Hydroxyglutaryl-CoA <=> Glutaconyl-CoA + H_2_O | **R03937** | **8.26** |
| 4 | Glutaconyl-CoA <=> Crotonyl-CoA + CO_2_ | **R03028** | **8.26** |
| 5 | Crotonyl-CoA + **NADPH** + H^+^ <=> Butanoyl-CoA + **NADP**^+^ | R09738 | - |
|  | Crotonyl-CoA + **NADH** + H^+^ <=> Butanoyl-CoA + **NAD**^+^ | **R01171** | **8.26** |
|  | Crotonyl-CoA + **FADH_2_** + H^+^ <=> Butanoyl-CoA + **FAD** | R01175 | - |
| 6 | Butanoyl-CoA + **NADPH** + H^+^  <=> Butanal + CoA + **NADP^+^** | R01173 | - |
|  | Butanoyl-CoA + **NADH** + H^+^ <=> Butanal + CoA + **NAD^+^** | **R01172** | **8.26** |
| 7 | Butanal + **NADH** + H^+^ <=> n-Butanol + **NAD^+^** | **R03544** | **8.26** |
|  | Butanal + **NADPH** + H^+^ <=> n-Butanol + **NADP^+^** | R03545 | - |

These values were obtained maximizing butanol production in anaerobic conditions with the glucose uptake rate set to 10 mmol.(g_DW_.h)^-1^. For each reaction from the heterologous pathway added to *i*JO1366 model the respective flux (mmol.(g_DW_.h)^-1^) is shown. DW: Dry Weight.
