# Supplementary material for: Discovery and implementation of a novel pathway for n-butanol production via 2-oxoglutarate
Source: Biotechnol Biofuels. 2019 Sep 30;12:230. doi: 10.1186/s13068-019-1565-x (PMC6767645; doi:10.1186/s13068-019-1565-x)
Supplement: Supplementary file 2 — Additional file 2: Fig. S1. Medium formulation influence on butanol accumulation. [file 13068_2019_1565_MOESM2_ESM.docx]

### Additional File 2: Medium formulation influence on butanol accumulation

The medium composition is one of the major aspects considered during the optimization of a bioprocess. In order to determine if the medium formulation used for the initial tests (Fig 2) could be further optimized, we omitted some of the extra components added: the extra amino acid mixture (added to improve protein expression), riboflavin and iron (III) citrate (added to improve the activity of the 2-hydroxyglutarate dehydratase) and glutamate (added to increase the availability of 2-oxoglutarate). The results obtained from different medium formulations are depicted in Figure S1.


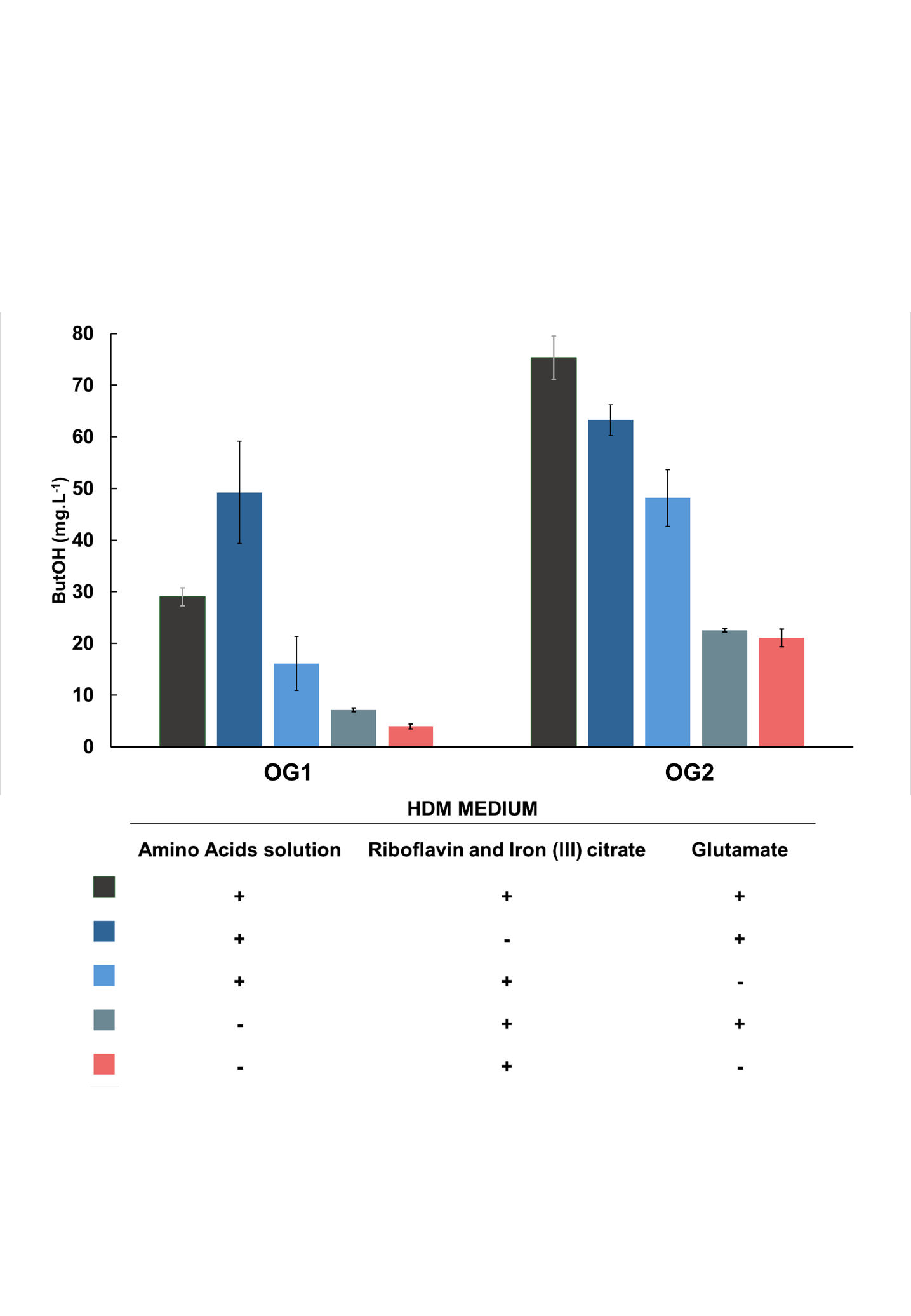


**Fig. S1 *Medium formulation influence on butanol accumulation.*** *Final extracellular butanol titer (mg.L^-1^) for strains OG1 and OG2 for different formulations of the HDM medium. Cells were grown in shake-flasks at 37 °C until 0.4-0.5 OD600, and then 0.5 mM of IPTG was added. Data are shown as mean ± S.D. from three independent experiments. ButOH – Butanol.*
