# Supplementary material for: Discovery and implementation of a novel pathway for n-butanol production via 2-oxoglutarate
Source: Biotechnol Biofuels. 2019 Sep 30;12:230. doi: 10.1186/s13068-019-1565-x (PMC6767645; doi:10.1186/s13068-019-1565-x)
Supplement: Supplementary file 3 — Additional file 3: Table S2. Reactions added to Escherichia coli genome scale metabolic model iJO1366. [file 13068_2019_1565_MOESM3_ESM.docx]

**Additional file 3: Reactions added to the model**

**Table S2 Reactions added to *Escherichia coli* Genome Scale Metabolic Model *i*JO1366.**

| Reaction | ID | Reaction |
| --- | --- | --- |
| 1 | R03534 | (R)-2-Hydroxyglutarate + FAD <=> 2-Oxoglutarate + FADH_2_ |
|  | R08198 | (R)-2-Hydroxyglutarate + NAD^+^ <=> 2-Oxoglutarate + NADH + H^+^ |
| 2 | R04000 | Acetyl-CoA + (R)-2-Hydroxyglutarate <=> Acetate + (R)-2-Hydroxyglutaryl-CoA |
| 3 | R03937 | Glutaconyl-CoA + H_2_O <=> (R)-2-Hydroxyglutaryl-CoA |
| 4 | R03028 | Glutaconyl-CoA <=> Crotonyl-CoA + CO_2_ |
| 5 | R09738 | Butanoyl-CoA + NADP^+^ <=> Crotonyl-CoA + NADPH + H^+^ |
|  | R01171 | Butanoyl-CoA + NAD^+^ <=> Crotonyl-CoA + NADH + H^+^ |
| 6 | R01173 | Butanal + CoA + NADP^+^ <=> Butanoyl-CoA + NADPH + H^+^ |
|  | R01172 | Butanal + CoA + NAD^+^ <=> Butanoyl-CoA + NADH + H^+^ |
| 7 | R03544 | Butanal + NADH + H^+^ <=> Butanol_c + NAD^+^ |
|  | R03545 | Butanal + NADPH ^+^ H^+^ <=> Butanol_c + NADP^+^ |
| Drain | R_EX_C06142_ | Butanol_c<=> Butanol_e |

These reactions correspond to the different catalytic steps of the most promising heterologous pathway to produce butanol, as well as drain to excrete this compound. Some of those steps have alternative cofactors.
