# Supplementary material for: Discovery and implementation of a novel pathway for n-butanol production via 2-oxoglutarate
Source: Biotechnol Biofuels. 2019 Sep 30;12:230. doi: 10.1186/s13068-019-1565-x (PMC6767645; doi:10.1186/s13068-019-1565-x)
Supplement: Supplementary file 4 — Additional file 4: Table S3. Sequences of primers used in the cloning procedures of this study; Table S4. List of plasmids used or designed in this study. Table S5. List of strains and genomic DNA used or engineered for this study. [file 13068_2019_1565_MOESM4_ESM.docx]

## Additional file 4: Genes, Plasmids and Primers

*E. coli* NEB 5-alpha cells were used for gene cloning and vector propagation. These strains were cultured in LB with the appropriate antibiotics concentration. All cultivations were performed at 37 °C and, in the case of liquid cultures, under shaking conditions (200 rpm).

For long-term storage, glycerol was added to a final concentration of 30 % (v/v) to overnight cultures in selective media and kept in a -80 °C freezer.

*hgdH*, *gcdH*, *hgdABC* and *gctAB* genes were codon-optimized through ATGenium for *E. coli*, synthesized and cloned in pHTP0 vector by NZYTech (Lisbon, Portugal). *gcdA* was amplified from *Clostridium symbiosum* ATCC 14940 genomic DNA obtained from DSMZ (Braunschweig, Germany). The *adhE* and *thl* genes were amplified from template plasmid pmTA1 (43). The genes *hbd* and *crt* were amplified using the plasmid prBCS (43) as template. The gene *atoB* was amplified using as template genomic DNA from *Escherichia coli* K12 (MG1655). The gene *ter* from *Treponoema denticola* (NCBI Sequence ID: AE017226.1) was synthesized by ATG:biosynthesis (Freiburg, Germany) .

Compatible vectors pETDuet-1 pCDFDuet-1, pCOLADuet-1 and pRSFDuet-1 (Novagen, Darmstadt, Germany) were used to provide individual expression of each protein under the control of the T7lac promoter and a ribosome binding site (RBS).

In Table S3 the primers used in this study for PCR amplification are shown.

**Table S3 Sequences of primers used in the cloning procedures of this study**

| Primer | Sequence | Restriction Sites * |
| --- | --- | --- |
| *adhE_fw* | CCGAATTCATGAAAGTCACAACAGTAAAGG | *EcoR*I |
| *adhE_rev* | CCGCGGCCGCTTAAGGTTGTTTTTTAAAACAATT | *Not*I |
| *ter_fw* | CCCATATGATTGTAAAACC | *Nde*I |
| *ter_rev* | CCCTCGAGTTAAATC | *Xho*I |
| *hgdABC_fw* | CCGAGCTCATGAGTATCTATACCCTGGGC | *Sac*I |
| *hgdABC_rev* | CCGCGGCCGCTTATTTTTGCATCTCCAAAAC | *Not*I |
| *gcdH_fw* | CCCATATGGCAACCAAAGCAAG | *Nde*I |
| *gcdH_rev* | CCCTCGAGTCAAAAGAACGCTTGAATACC | *Xho*I |
| *hgdH_fw* | CCGGTACCATGAAAGTGCTGTGCTACGG | *Kpn*I |
| *hgdH_rev* | CCCTCGAGTTATTTGATTTTGTTCGGGC | *Xho*I |
| *gctAB_fw* | CCGGATCCATGAGCAAAGTCATGACCC | *BamH*I |
| *gctAB_rev* | CCAAGCTTTTATTTGGCTTCAGTTGGAAC | *Hind*III |
| *gcdA_fw* | ccCATatgaatatgtattcaatgccaggat | *Nde*I |
| *gcdA_rev* | ccCTCGAGttatttaccaaatgtctcgaattcacg | *Xho*I |
| *thl_fw* | CCGGATCCATGAGAGATGTAGTAATAGTAAGTGC | *BamH*I |
| *thl_rev* | CCAAGCTTTTAGTCTCTTTCAACTACGAGAGC | *Hind*III |
| *atoB_fw* | CCGGATCCATGAAAAATTGTGTCATCGTCAG | *BamH*I |
| *atoB_rev* | CCAAGCTTTTAATTCAACCGTTCAATCACC | *Hind*III |
| *hbd_fw* | CCGGATCCATGAAAAAGGTATGTGTTATAG | *BamH*I |
| *hbd_rev* | CCGAGCTCTTATTTTGAATAATCGTAGAAAC | *Sac*I |
| *crt_fw* | CCGGTACCATGGAACTAAACAATGTC | *Kpn*I |
| *crt_rev* | CCCTCGAGCTATCTATTTTTGAAGCCTT | *Xho*I |

(*restriction sites are underlined). fw – forward; rev – reverse.

The success of the plasmid constructions was confirmed by sequencing the regions of interest with the appropriate primers. In Table S4, the plasmids used or constructed in this study, as well as the respective major features are depicted.

**Table S4** **List of plasmids used or designed in this study.**

| Plasmid | Features | Source |
| --- | --- | --- |
| pETDuet-1 | ColE1(pBR322) ori, lacI, double T7lac, Amp^R^ | Novagen |
| pCDFDuet-1 | CloDF13 ori, lacI, double T7lac, Strep^R^ | Novagen |
| pRSFDuet-1 | RSF ori, lacI, double T7lac, Kan^R^ | Novagen |
| pCOLADuet-1 | COLA (ColA) ori, double T7lac, Kan^R^ | Novagen |
| pETDuet_adhE_ter | pETDuet-1 carrying *adhE*1 from *C. acetobutylicum* and *ter* from *T. denticola* | This study |
| pCDFDuet_gctAB_hgdH | pCDFDuet-1 carrying *gctAB* and *hgdH* from *A. fermentans* | This study |
| pRSFDuet_gcdH_hgdABC | pRSFDuet-1 carrying *hgdC* from *A. fermentans*; *hgdAB* from *Clostridium symbiosum* and *gcdH* from *Pseudomonas aeruginosa PAO1* | This study |
| pCOLADuet_gcdA_hgdABC | pCOLADuet-1 carrying *hgdC* from *A. fermentans*; *hgdAB* from *Clostridium symbiosum* and *gcdA* from *C. symbiosum* | This study |
| pCOLADuet_hbd_crt | pCOLADuet-1 carrying *hbd* and *crt* from *C. acetobutylicum* | This study |
| pCDFDuet_thl | pCDFDuet^-^1 carrying *thl* from *C. acetobutylicum* | This study |
| pCDFDuet_atoB | pCDFDuet-1 carrying *atoB* from *E. coli* | This study |
| pmTA1 | Gm^r^, lacI, taclac: *thil*, *adhE1* | (43) |

| prBCS | Tet^r^, lac: BCS (crt, bcd, etfAB, hbd) | (43) |
| --- | --- | --- |

### *Bacterial strains*

*E. coli* K12 MG1655 (DE3) and *E. coli* BL21 (DE3) were used as hosts for gene expression under control of the T7 promotor.

The strains here used were obtained by transforming *E. coli* BL21 (DE3) and *E. coli* K12 MG1655 (DE3) with the indicated plasmids by electroporation using 0.1 cm-gap electroporation cuvettes at a voltage of 1.8 KV. Electrocompetent cells were prepared using the protocol developed by (59). Positive transformants were isolated in LB agar plates, containing the appropriate antibiotic concentrations and incubated at 37 °C, overnight. To confirm the success of the transformation, a few transformant colonies were cultivated in LB liquid medium with antibiotics, overnight. After, plasmids were extracted and digested with appropriate restriction enzymes. The correct fragment lengths were confirmed by running the digestion in a 1 % (w/v) agarose gel.

Table S5 summarizes the strains of *E. coli* used or engineered for this study.

**Table S5 List of strains used or engineered for this study.**

| Strains | Relevant genotype | Source |
| --- | --- | --- |
| *E. coli* K12 MG155 (DE3) | F - λ - ilvGrfb- 50 rph- 1 λ(DE3) | (43) |
| *E. coli* BL21 (DE3) | fhuA2 [lon] ompT gal (λ DE3) [dcm] ∆hsdS  λ DE3 = λ sBamHIo ∆EcoRI-B int::(lacI::PlacUV5::T7 gene1) i21 ∆nin5 | New England Labs |
| OG1 | *E. coli* BL21 DE3 pETDuet_adhE_ter; pCDFDuet_gctAB_hgdH; pRSFDuet_gcdH_hgdABC | This study |
| OG2 | *E. coli* K12 MG1655 DE3 pETDuet_adhE_ter; pCDFDuet_gctAB_hgdH; pRSFDuet_gcdH_hgdABC | This study |
| OG3 | *E. coli* BL21 DE3 pETDuet_adhE_ter; pCDFDuet_gctAB_hgdH; pRSFDuet_gcdA_hgdABC | This study |
| OG4 | *E. coli* K12 MG1655 DE3 pETDuet_adhE_ter; pCDFDuet_gctAB_hgdH; pCOLADuet_gcdA_hgdABC | This study |
| CT_OG1 | *E. coli* BL21 DE3 pETDuet_adhE_ter; pRSFDuet_gcdH_hgdABC | This study |
| CT_OG2 | *E. coli* K12 MG1655 DE3 pETDuet_adhE_ter; pRSFDuet_gcdH_hgdABC | This study |
| ACT1 | *E. coli* BL21 DE3 pCDFDuet_thl; pCOLADuet_hbd_crt; pETDuet_adhE_ter | This study |
| ACT2 | *E. coli* K12 MG1655 DE3 pCDFDuet_thl; pCOLADuet_hbd_crt; pETDuet_adhE_ter | This study |
| ACT3 | *E. coli* BL21 DE3 pCDFDuet_atoB; pCOLADuet_hbd_crt; pETDuet_adhE_ter | This study |
| ACT4 | *E. coli* K12 MG1655 DE3 pCDFDuet_atoB; pCOLADuet_hbd_crt; pETDuet_adhE_ter | This study |
